# Cold atmospheric plasma and silymarin nanoemulsion synergistically inhibits human melanoma tumorigenesis via targeting HGF/c-MET downstream pathway

**DOI:** 10.1186/s12964-019-0360-4

**Published:** 2019-05-24

**Authors:** Manish Adhikari, Neha Kaushik, Bhagirath Ghimire, Bhawana Adhikari, Sanjula Baboota, Abdulaziz A. Al-Khedhairy, Rizwan Wahab, Su-Jae Lee, Nagendra Kumar Kaushik, Eun Ha Choi

**Affiliations:** 1 Plasma Bioscience Research Center, Applied Plasma Medicine Center, Department of Electrical and Biological Physics, Kwangwoon University, Seoul, Republic of Korea; 2 Department of Life Science, Hanyang University, Seoul, 04763 Republic of Korea; 3 Department of Pharmaceutics, School of Pharmaceutical Education and Research, Jamia Hamdard, Delhi, India; 4 Zoology Department, College of Science, King Saud University, Riyadh, Saudi Arabia; 5 Al-Jeraisy, Chair of DNA Research, College of Science, Riyadh, Saudi Arabia

**Keywords:** Non thermal plasma, Silymarin nanoemulsion, Melanoma, HGF/c-MET, Cancer Stemness, Epithelial-mesenchymal transition

## Abstract

**Background:**

Recent studies claimed the important role of cold atmospheric plasma (CAP) with nanotechnology in cancer treatments. In this study, silymarin nanoemulsion (SN) was used along with air CAP as therapeutic agent to counter human melanoma.

**Methods:**

In this study, we examined the combined treatment of CAP and SN on G-361 human melanoma cells by evaluating cellular toxicity levels, reactive oxygen and nitrogen species (RONS) levels, DNA damage, melanoma-specific markers, apoptosis, caspases and poly ADP-ribose polymerase-1 (PARP-1) levels using flow cytometer. Dual-treatment effects on the epithelial–mesenchymal transition (EMT), Hepatocyte growth factor (HGF/c-MET) pathway, sphere formation and the reversal of EMT were also assessed using western blotting and microscopy respectively. SN and plasma-activated medium (PAM) were applied on tumor growth and body weight and melanoma-specific markers and the mesenchymal markers in the tumor xenograft nude mice model were checked.

**Results:**

Co-treatment of SN and air CAP increased the cellular toxicity in a time-dependent manner and shows maximum toxicity at 200 nM in 24 h. Intracellular RONS showed significant generation of ROS (< 3 times) and RNS (< 2.5 times) in dual-treated samples compared to control. DNA damage studies were assessed by estimating the level of γ-H2AX (1.8 times), PD-1 (> 2 times) and DNMT and showed damage in G-361 cells. Increase in Caspase 8,9,3/7 (> 1.5 times), PARP level (2.5 times) and apoptotic genes level were also observed in dual treated group and hence blocking HGF/c-MET pathway. Decrease in EMT markers (E-cadherin, YKL-40, N-cadherin, SNAI1) were seen with simultaneously decline in melanoma cells (BRAF, NAMPT) and stem cells (CD133, ABCB5) markers. In vivo results showed significant reduction in SN with PAM with reduction in tumor weight and size.

**Conclusions:**

The use of air CAP using μ-DBD and the SN can minimize the malignancy effects of melanoma cells by describing HGF/c-MET molecular mechanism of acting on G-361 human melanoma cells and in mice xenografts, possibly leading to suitable targets for innovative anti-melanoma approaches in the future.

**Electronic supplementary material:**

The online version of this article (10.1186/s12964-019-0360-4) contains supplementary material, which is available to authorized users.

## Background

Modern developments in molecular oncology opened new therapeutic approaches that target the key effectors of the pathways responsible for the pathogenesis of melanoma. Some examples are activation of the neuroblastoma RAS viral oncogene homolog (NRAS), the v-raf murine sarcoma viral oncogene homolog B1 (BRAF), or the cell surface-mesenchymal-epithelial transition (c-MET) or the suppression of the antitumor immune response by certain immune regulatory molecules and processes, such as T-lymphocyte-associated antigen 4 (CTLA4) and programmed cell death 1 (PD-1) [1]. Following the traditional type of therapy, the average survival time of patients with metastatic melanoma is estimated to be approximately 6–12 months. With the five-year survival rate at less than 10% in most cases [2]. Therefore, there is an urgent need to develop efficient and cost-effective remedial agents that can be applied as part of a treatment for melanoma. Currently, cold atmospheric plasma (CAP) is an emerging biomedical technique used as a selective cancer treatment [3]. CAP essentially refers to a cocktail containing reactive oxygen and nitrogen species (RONS), ultraviolet rays (UV), and charged particles, the combination of which induces physical and chemical changes to the biological surfaces [4]. Presently, CAP is used for wound healing, tissue regeneration and inert surface sterilization [5, 6]. Previous studies have shown that CAP can kill cancer cells and significantly minimize solid tumor sizes with minimal damage to normal cells. Apart from the ameliorative activity of CAP, nanotechnology has dramatically influenced drug delivery research to improve the therapeutic performance capabilities of drugs as part of the effort to cure different cancers [7]. In present melanoma reduction studies, a nanoemulsion was prepared from a well-known herb known as silymarin (SN) which use worldwide as a hepatoprotective agent and shows applications in cancer therapies. It has a natural hydrophobic structure with low water solubility and bioavailability. Hence, the formulation was prepared as per our previous publication [8] which involve a heterogeneous dispersion of two immiscible liquids (oil-in-water) has been used to improve hydrophobic drug delivery and minimize metabolic drug degradation, exhibiting sustained and triggered releases of drugs [9]. SN has been reported to kill hepatocarcinoma cells and nephron cells using γ-radiation [8, 10]. Over the last few years, the combination of nanoparticle and plasma technology has shown promise in plasma medicine, particularly in cancer therapy by halting the activation of the PI3/AKT pathway [11, 12]. Therefore, the combination of CAP and nanoparticles/nanoemulsion could contribute to improving the selective permeability of membranes by CAP-generated RONS species, leading to the intracellular diffusion of nanoparticles towards diseased sites within tissues [13].

Henceforth, the focus of this study is to develop a novel anti-tumorigenic therapeutic method by integrating CAP using a μ-dielectric barrier discharge (μ-DBD) CAP device with a silymarin nanoemulsion (SN) and then to evaluate its underlying mechanism with regard to targeted melanoma treatment. The results demonstrate the down-regulation of metabolic viability and melanoma- and melanoma-stem-cell-specific markers. A new approach toward abrogating the HGF/c-MET signaling pathway using CAP and the SN could provide a novel strategy during the treatment of melanoma. In vivo studies substantiate and support our in vitro results given the reduced tumor sizes and tumor weights after the co-treatment. Hence, these results present evidence of a plausible potential role of the CAP for improving targeted SN delivery ultimately to inhibit the progress of melanoma.

## Methods

### Chemicals and antibodies

Silymarin, Solutol® HS 15, MTT (3-(4,5-dimethylthiazol-2-yl)-2,5-diphenyltetrazolium bromide), dimethyl sulphoxide, epithelial growth factor (EGF), basic fibroblast growth factor (bFGF), β-actin, annexin V-PI kit, nestin and Ki-67 were purchased from Sigma-Aldrich, USA. RPMI-1640, DMEM F-12, phosphate-buffered saline, and the penicillin-streptomycin antibody cocktail solution used here were obtained from Welgene, Korea. Labrafac lipophile WL 1349 and Transcutol® HS 15 were procured from Gattefossé (France). H2DCFDA and DAFFM-DA were purchased from Molecular Probes, USA. Antibodies specific to c-Met, BRAF, YKL-40, N-cadherin, E-cadherin, and ABCB and PARP ELISA kits and anti-BRAF antibody were purchased from Abcam, Korea. FBS, Trypsin was procured from Hyclone (GE Healthcare Life Sciences). Anti-gamma H2AX antibody, anti-PD1 antibody and anti-CD133 antibody were obtained from BD Biosciences, USA. Anti-NAMPT antibody and anti-DNMT antibody were purchased by Novus Biological, USA. Caspase 3/7, 8 and 9 kits were obtained by Promega Corporation, USA. B27 supplement was purchased from Invitrogen and the antibody cocktail used in this study (penicillin & streptomycin) was procured from Gibco, Korea. Antibodies specific to PARP, Bcl-2, caspase-3, BAX, p53, p-c-Met (Tyr1234/1235), p-BRAF, p-AKT (T308), p-AKT(S473), AKT, and SNAI1 were procured from Cell Signaling Technology, USA. All primers were designed and purchased from DNA Macrogen, Korea.

### Cell culture

G-361 cells and SK-MEL-5 were cultured in RPMI-1640 cell culture media supplemented with 10% fetal bovine serum, 100 U/ml penicillin, and 100 mg/ml streptomycin and were maintained at 37^ο^C in a 5% humidified CO_2_ environment. The cells were routinely tested for mycoplasma test (MycoAlert™ Mycoplasma Detection Kit, Lonza, Switzerland). The passage number for G-361 cells was 30 and SK-MEL-5 was 20 at the time of experiments. For co-culturing with SN, G-361 and SK-MEL-5 cells were placed separately for 4 h in the SN before the CAP. For the spherical cultures, the sphere permissive medium was achieved through serum-free DMEM-F12 supplemented with 20 ng/ml EGF, bFGF, B-27 supplement (50X), and antibiotics (100 U/ml penicillin and 100 μg/ml streptomycin containing an antimycotic solution).

### μ-Dielectric barrier discharge (DBD) CAP device and irradiation

A CAP micro-dielectric barrier discharge (μ-DBD) surface CAP device consisting of electrodes with a silicon dioxide (SiO_2_) dielectric layer (30 mm) and hydration-prevention layers consisting of aluminum oxide (Al_2_O_3_) and a magnesium oxide (MgO) layer was used. The electrode gap was held at 200 µm. To generate the CAP, air was used as a feeder gas into the device at a flow rate of l.5 lpm and a 2-mm distance was maintained between the CAP source and the upper surface of the medium in the cell culture petri dish. No significant increase in the temperature of the treated media was observed after up to 5 min of CAP exposure. G-361 and SK-MEL-5 cells were seeded in culture dishes with a 35 mm diameter and treated for 300 s with μ-DBD CAP.

### Formulation of the SN and its characterization

The SN was fabricated according to the process outlined in our previous publication [8]. First, a pre-weighed amount of silymarin was dissolved in a calculated amount (15%) of oil (Labrafac Lipophile WL1349), a surfactant (50%) (Solutol HS 15) and a co-surfactant (35%) (Transcutol HP). The resulting emulsion was characterized in terms of the globule size, polydispersity index (PDI), and surface morphology and size by transmission electron microscopy (TEM). The globule size distribution and zeta potential were determined by photon correlation spectroscopy using a Zetasizer Nano ZS90 (Malvern Instruments, Worcestershire, UK; dynamic light scattering). The droplet size distribution was determined at a refractive index 1.41, viscosity of 5.0 PaS, and a dielectric constant 79.4.

### Metabolic cellular viability

The cytotoxic effects of CAP and the SN at different doses and time intervals on human melanoma G-361 and SK-MEL-5 cells were determined using a colorimetric MTT test which evaluates cellular metabolic activity based on the ability of mitochondrial succinate reductase to convert a yellow-colored dye (MTT) to purple formazan in living cells. Absorbance was recorded at 540 nm using a Synergy HT Biotek microplate reader. The metabolic activity is directly proportional to the tetrazolium reduction inside the cells and is calculated in terms of the percent of a control, which was arbitrarily assigned a value of 100% viability.

### Intracellular RONS measurement

Free radicals of ROS and RNS were estimated using the fluorescent dye H_2_DCFDA and DAFFM-diacetate dye, respectively. G-361 and SK-MEL-5 cells were cultured in six-well plates and treated with CAP and the SN respectively. H_2_DCFDA was added 30 min prior, whereas DAF-FM-diacetate was added 15 min before the completion of the incubation period of the cells. Both cells were then trypsinized and all measurements were completed within 30 min. RONS generation was conducted by flow cytometry and the outcome was quantitatively measured using FACS Suite software (Becton Dickinson and Co., Franklin Lakes, NJ, USA).

### Cellular damage studies

The induction and damage of DNA double-strand breaks (DSBs) in terms of γ-H2AX positive cells were analyzed in G-361 cells. The cells were harvested in the RPMI-1640 medium with 10% FBS overnight and acquired 24 h after treatment as per the manufacturer’s recommended procedures (BD Biosciences) using a FACSVerse flow cytometer. The number of γ-H2AX positive foci in the G-361 cells was visualized by immunocytochemistry using a fluorescent microscope along with DAPI dye (Fig. 3a).

To determine the melanoma-specific nuclear proteins, G-361 cells were treated with CAP and the SN at the described time intervals. They were then harvested using trypsinization, washed with phosphate-buffered saline, and correspondingly incubated with anti-PD-1 (Programmed cell death protein 1) antibody and anti-DNMT (DNA-methyl transferase) antibody as per the manufacturer’s protocol. Samples were immediately analyzed using BD FACSVerse software of the FACS suite.

### Caspase activity

To assess caspases, G-361 cells were seeded into 96-well plates and the caspase 3/7, 8 and 9 activities were estimated by measuring the luminescence (Promega, USA). The Caspase kits provided in lyophilized form and were converted to a working solution, and the luminescence levels were evaluated for all groups.

### Sandwich ELISA assay for cleaved PARP

To check the level of cellular damage, G-361 cells were cultured in 96-well plates after the CAP and SN treatment by estimating the degree of cleaved PARP. The cells were washed, fixed, and treated with an antigen retrieval buffer before the addition of the PARP primary antibody. On the next day, after the washing of the primary antibody, the secondary antibody was diluted according to the standard procedure and finally read at 450 nm. Background subtraction was necessary and was done by adding 50 μl of Janus green, after which the absorbance recorded at 595 nm. A sandwich ELISA kit (Abcam) was used to evaluate the effect of the aforementioned group on the induction of apoptosis in the cells.

### Immunoblotting and q-PCR analysis

Cell lysates were prepared by extracting proteins with lysis buffer (40 mM Tris-HCl (pH 8.0), 120 mM NaCl, 0.1% Nonidet-P40) supplemented with protease inhibitors for western blotting. Proteins were separated by SDS-PAGE and then transferred to a nitrocellulose membrane (Amersham, IL). The membranes were blocked with 5% skim milk in Tris-buffered saline and incubated overnight with primary antibodies at 4 °C. Blots were developed using a peroxidase-conjugated secondary antibody, and proteins were visualized using enhanced chemiluminescence (ECL) procedures (Amersham, IL) according to the manufacturer’s protocol. Total RNA was extracted using Trizol (Invitrogen, USA), after which qRT-PCR was performed using a Biorad 2X SYBR green mix. Reactions were carried out in a Biorad thermal cycler (Biorad, Korea), and the results were expressed as the fold change calculated using the ΔΔCt method relative to a control sample. β-actin was used as an internal normalization control. All primers were purchased from DNA Macrogen, Korea.

### FITC Annexin V–propidium iodide (PI) staining for apoptosis assay

Apoptotic cell death was determined by staining the G-361 cells with Annexin V-PI. Cells were incubated in 35mm^2^ petri dishes after the CAP and SN treatment, washed with PBS, and then resuspended in annexin V binding buffer and incubated for 30 min at RT (room temperature). Subsequently, FITC-annexin V was added to each tube and the tubes were incubated for 20 min at RT. Propidium iodide (PI) was added to the cells with incubation for 20 min at RT, and cell apoptosis was analyzed by FACSVerse. Viable cells were negative for both PI and Annexin V; apoptotic cells were positive for Annexin V and negative for PI, whereas late apoptotic dead cells displayed both high Annexin V and PI labeling. Non-viable cells, which underwent necrosis, were considered as positive for PI and negative for Annexin V.

### Melanoma -specific markers

To check for the specificity of melanoma, G-361 cells were washed, trypsinized, and pelletized for both melanoma-specific markers. The cells were fixed with 100 μl of 3.7% formaldehyde and vortexed to create a single cell suspension. After washing with 1XPBS, cells were resuspended in pre-chilled methanol and incubated for 30 min at 4 °C. They were then rewashed with 1XPBS two times, after which anti-BRAF antibody and anti-NAMPT antibodies were added independently. Subsequently, after antibody treatments a final wash with 1XPBS were done before acquiring the samples using FACSVerse (BD Biosciences, USA).

### Migration and invasion assays

Polycarbonate filters (0.8 μm; Corning USA) were coated with a reconstituted growth-factor reduced matrigel (BD Biosciences). Afterward, 2 × 10^4^ cells in 200 μl of serum-free growth medium were seeded into the upper chamber. The cells were then incubated at 37 °C and allowed to migrate towards the complete growth medium for 24 or 48 h. Non-invading cells were removed using cotton swabs. For migration assays, inserts were not coated with the matrigel.

### Immunofluorescence

To visualize the N-cadherin expression and γ-H2AX foci, cells were fixed in 4% paraformaldehyde for 10–15 min and blocked with goat serum (Sigma-Aldrich) for 30 min, after which they were incubated at 4 °C overnight with appropriate primary antibodies (1:200). Following two washes with 1X PBS, the cells were incubated with a conjugated secondary antibody (1:1000; Invitrogen at RT for 2 h and counter-stained with 4′,6-diamidino-2-phenylindole (DAPI; Sigma) for approximately 10 min for nuclear staining. The fluorescence staining intensity and intercellular location were examined using a fluorescence-inverted microscope (Olympus BX51, Japan).

### Sphere culture and sphere-forming assay

For sphere-formation assays, the size of the spheres was determined using the Motic Images Plus 2.0 software in three randomly chosen visual fields until day 4. Clones were photographed using a phase-contrast microscope, and the sphere diameter was measured using the Motic Images Plus 2.0 software.

### Melanoma-stem-cell-specific markers

Expression of melanoma stem cell markers, were estimated by labeling G-361 cells with anti CD133 antibody (BD Biosciences, USA) or anti ABCB5 antibody (Abcam, Korea). All samples were incubated for 20 min at 4 °C, washed twice with PBS, and immediately analyzed using a BD FACSVerse cytometer and the FACS suite software.

### Tumor xenografts in nude mice

G-361 cells (human melanoma, 1 X 10^6^ cells/200 μl PBS) were injected intradermally into the upper right flank of nude male mice (CAnN.Cg-Foxn1/ 5 weeks of age; Orientbio, Korea). The mice were randomly divided into vehicle, PAM only, SN only and co-treatment groups after achieving a tumor size of 100mm^3^. Plasma activated media (PAM) were freshly prepared because of reduction in free radicals, with a 10-min CAP treatment of incomplete RPMI media using μ-DBD CAP. Mice were treated with 1 mg/kg of body weight of the SN 1 day before the PAM treatment to the co-treatment group. The vehicle group of mice received 200 μl of PAM once for each of the next 3 days by an intradermal injection into the tumor, and the control group received the same volume of RPMI medium into the right flank. Body weights were estimated on each subsequent day after the injections of the tumors, and tumor sizes were measured with Vernier calipers (calculated volume = shortest diameter X longest diameter/2) at two- day intervals. This study was reviewed and approved by the Institutional Animal Care and Use Committee (IACUC) of the Center for Laboratory Animal Sciences, Medical Research Coordinating Center.

### Immunohistochemistry

Tumor tissues were excised with proper precautions and fixed in formalin for the preparation of paraffin sections. Paraffin-embedded tumor sections were deparaffinized in xylene and then with 100, 90, 80 and 70% ethanol, followed by phosphate-buffered saline (PBS). Tumor sections were stained with hematoxylin and eosin (H & E) or immunostained overnight at 4^ο^C with the BRAF antibody (1:300; Abcam), the cMET (1:300; Abcam), the CD133 (1:200; Abcam) antibody, and Ki67 (1:200; Abcam). After washing in PBS, a 1:200 dilution of biotinylated goat anti-rabbit IgG or anti-mouse IgG antibody in a blocking solution was applied to the sections and they were incubated for 30–40 min. Following the PBS treatment, ABC reagent was applied to the sections and they were incubated a further 30 min. Color reaction tests were performed with 3, 30-diaminobenzidine (Vector Laboratories) and the slides were washed twice with PBS. After hematoxylin counterstaining and clearing with a graded ethanol series and xylene, sections were mounted with Canada balsam. Images were photographed using an IX71 microscope (Olympus) equipped with the DP71 digital imaging system (Olympus).

### Statistical analysis

Data were expressed as the means ± SD of triplicates. The statistical significance of the difference between the values of the control and treatment groups was determined using Student’s t-tests, and in each case the levels of significance are indicated as ^*^, *p* < 0.05; ^δ^, *p* < 0.01; and ^#^, *p* < 0.001.

## Results

### Characterization of CAP and SN

**Fig. 1:**
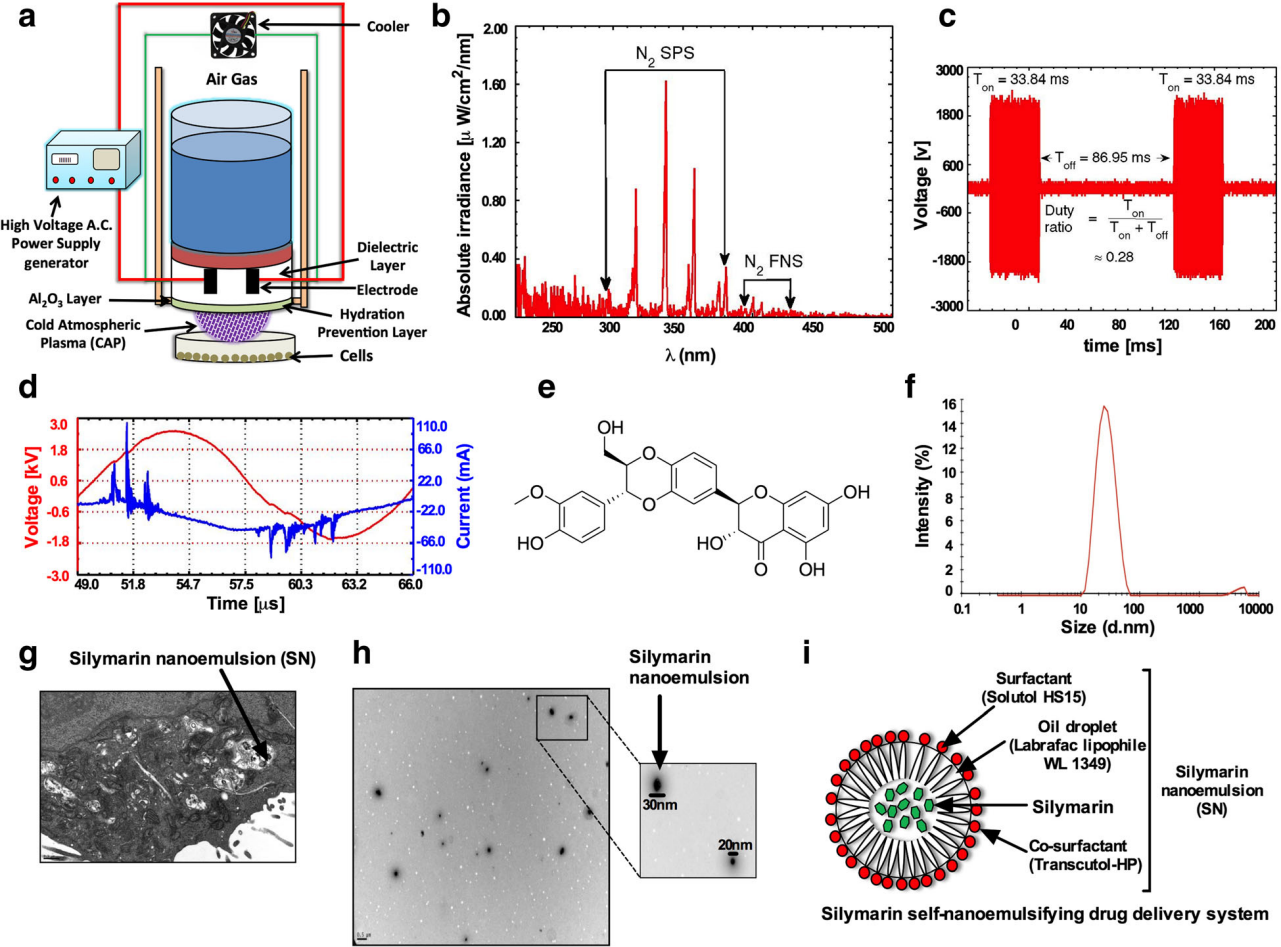
Description of μ-DBD air CAP and SN. (**a**) Schematic representation of the μ-DBD air CAP device and experimental setup used for the CAP treatment. (**b**) Optical emission spectrum (OES) composition of the air μ-DBD CAP source between 200 and 500 nm. (**c**) The μ-DBD device on time (T_on_) and off time (T_off_). (**d**) Voltage and current waveforms for one cycle of the T_on_ period of the μ-DBD air CAP device. (**e**) Chemical structure of the silymarin. (**f**) Globule size distribution of the SN. (**g**) Transmission electron microscopy (TEM) image of the SN inside G-361 melanoma cells. (**h**) SN size estimation using ultra-high-magnification TEM. (**i**) Schematic representation of the constituents of SN

The μ-DBD CAP used here with air gas at a flow rate of 1.5 lpm (Fig. 1a) with custom-built inverter operated in “dimming mode” which interacts with the liquid culture media to produce reactive species. Measurement outcomes of the spectral composition of the DBD source between 200 and 500 nm (Fig. 1b). The spectra are dominated mostly by emissions from the nitrogen second positive system (N_2_ SPS) at wavelengths of 296 nm, 316 nm, 337 nm, and 358 nm in addition to nitrogen first negative system (N_2_ FNS) around 400 nm. There is also weak emission from hydroxyl radicals at 309 nm [14]. The on-time (T_on_) and off-time (T_off_) values of the source were set to 33.84 ms and 86.95 ms, respectively, with a duty ratio of 26% (Fig. 1c). The applied voltage (rms), current (rms), frequency and energy required to sustain the discharge were 1.33 kV, 12 mA, 58 kHz, and 6.81 mJ/cm^2^, respectively (Fig. 1d). Another figure (Fig. 1e) shows the structure of the silymarin which is the parent compound for making SN and was prepared by varying and fixing the concentration of the selected oil, surfactant and co-surfactant (data not present) [8]. The average particle size and polydispersity index were found to be 25.53 d.nm diameter and 0.169 ± 0.01, respectively, indicating a nano-range with a minimum variation of the SN size (Fig. 1f). The concentration of SN refers to the amount of nanosilymarin (1 mg/ml) present along with labrafac lipophile WL 1349, solutol HS and transcutol HP.

### Surface morphology and SN uptake analysis

To assess the surface morphology, SN (diluted 500 times) internalization in the G-361 cells was visualized by transmission electron microscopy (TEM-JEOL 10/10 transmission electron microscope, CA, USA) (Fig. 1g). The SN consisted of discrete, spherical oil globules with diameters ranging from 20 to 30 nm. Taken together, the TEM data suggest that the surface morphology and estimated size of the SN were nearly identical to the results confirmed by the Zetasizer (Fig. 1g, h). The representation for SN is described in Fig. 1i.

### Cytotoxicity and intracellular RONS estimation in melanoma cells

**Fig. 2:**
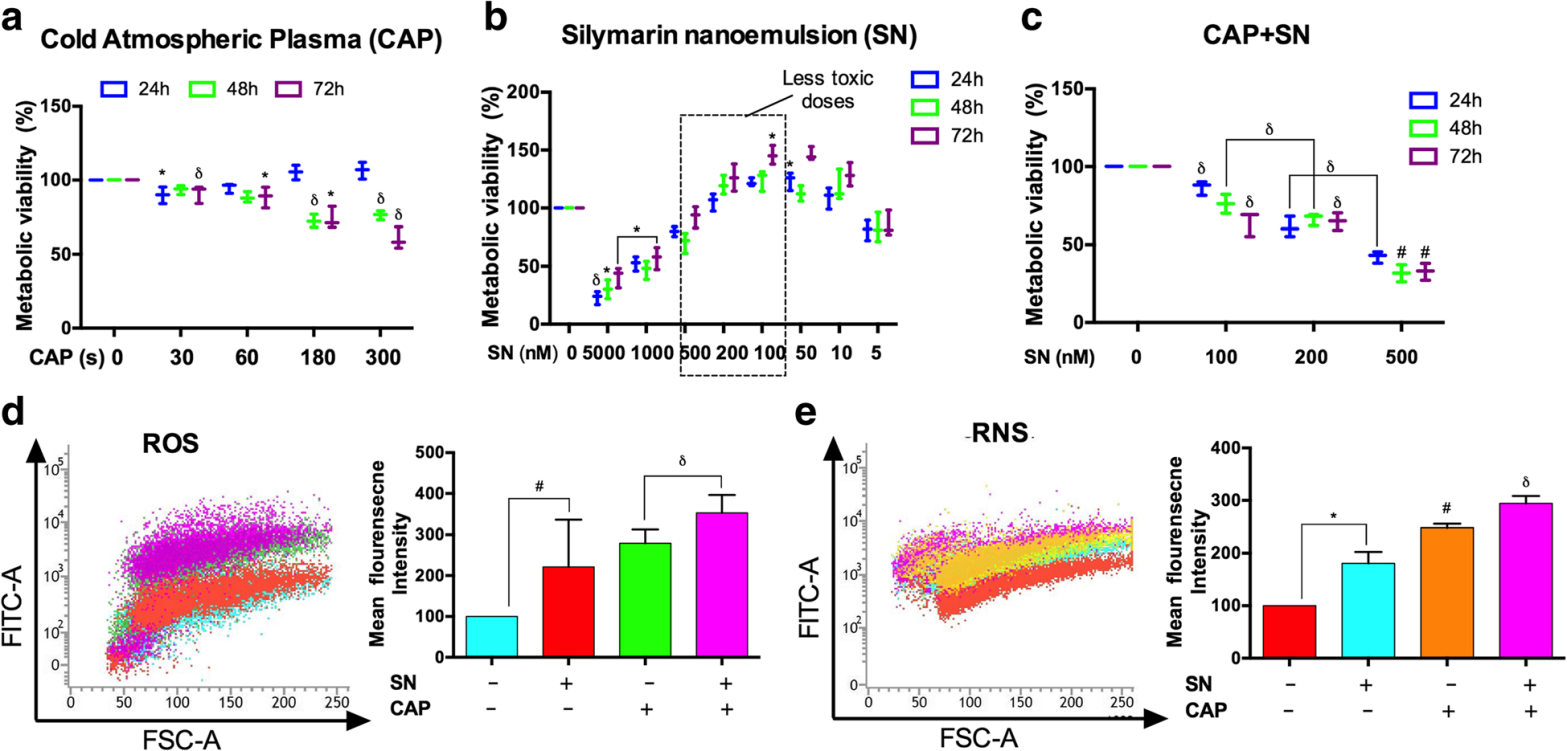
Total cellular damage effects of the SN and CAP treatment of G-361 cells. Effect on metabolic viability according to MTT assays at (**a**) different CAP treatment time intervals, (**b**) using SN at different concentrations (**c**) Incubation of SN for 4 h prior to the CAP treatment at different time intervals. (**d**) ROS measurement using H2DCF-DA and (**e**) RNS measurement using DAF-FM-DA dye by flow cytometry with SN (100 nM), CAP (180 s), and the co-treatment of CAP and SN. Student’s t-tests were performed, and the levels of significance are indicated as follows: ^*^, *p* < 0.05; ^δ^, *p* < 0.01; and ^#^*, p < 0.001*

MTT assay revealed that the cellular viability was significantly decreased in a time- and concentration-dependent manner in G-361 cells (Fig. 2a). However, SK-MEL-5 cells showed a significant increase in metabolic viability at 30 s time-interval which decreased further when received CAP treatment for 180 s and 300 s respectively (Additional file 1: Figure S1A). We also noted that CAP (< 180 s) alone did not efficiently attenuate cell viability levels, whereas SN alone increased the metabolic viability rate to 100 nM in G-361 cells. Consequently, the optimal concentration for the enhancement of the cellular toxicity was considered to be 100 nM, beyond which significant changes were not noted, remaining constant for all experiments (Fig. 2b, c).

SN interaction with SK-MEL-5 cells are almost similar compared to G-361 cells and it showed maximum viability at 100 nM SN concentration. However, combination treatment of SN and CAP didn’t reveal much toxicity on SK-MEL-5 cells at 100 nM treatment with 180 s though, it decreased further at 200 nM and 500 nM but didn’t get IC50 at any treatment–time (Additional file 1: Figure S1B, C). Therefore, we choose G-361 cells for our further studies. The level of RONS, with the dichlorofluorescein, mean fluorescence intensity was assessed at 24 h post-incubation using the aforementioned dose, where the ROS activity was dramatically increased after CAP treatment, SN treatment and co-treatment group in G-361 cells (Fig. 2d). The results indicate that the co-treatment with the CAP and SN simultaneously induced a three-fold increase in ROS generation (by DCFDA) and significantly a two-fold increase in RNS (by DAF-FM) fluorescence as compared to the control samples in G-361 cells (Fig. 2e).

### DNA damage through γ-H2AX and PD-1 expression in melanoma cells

**Fig. 3:**
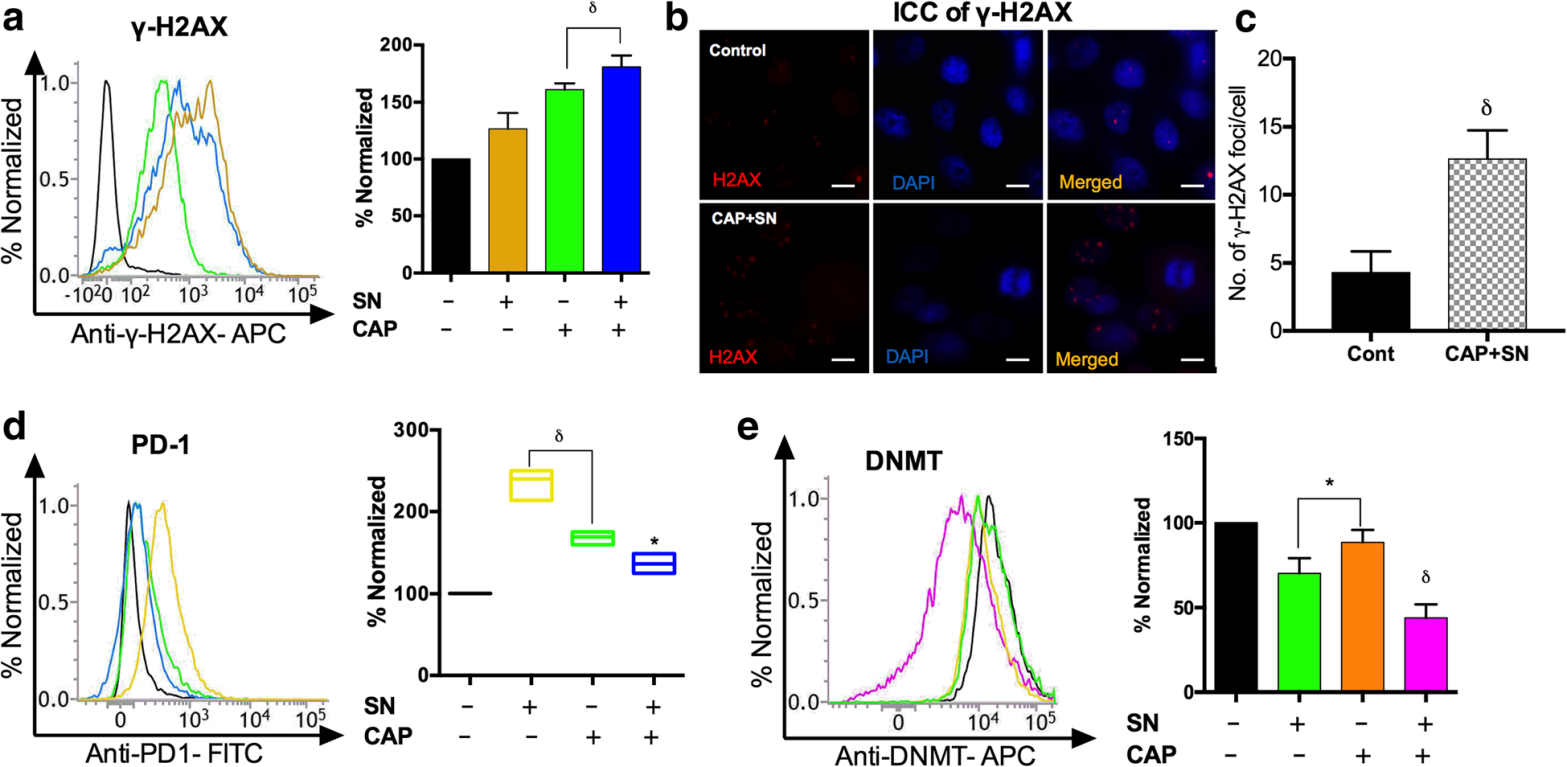
SN (100 nM) and CAP (180 s)-mediated cellular damage studies of G-361 cells. (**a**) estimating the level of the DNA damage by γ-H2AX marker by flow cytometry (**b**) Immunocytochemistry (ICC) of γ-H2AX (Red) showing DNA damage by a co-treatment of CAP and SN and counterstaining with DAPI (Blue) to fix cell imaging to visualize the nuclei, using fluorescence microscopy (40X magnification). The scale bar corresponds to 10 mm. (**c**) Graphical representation of the number of γ-H2AX foci in control and co-treated groups. (**d**) Cell surface marker PD-1 level quantified using flow cytometry. (**e**) Melanoma-specific marker DNMT (DNA methyltransferase) was measured using flow cytometry. Student’s t-tests were performed, and the levels of significance are indicated as follows: ^*^, *p* < 0.05; ^δ^, *p* < 0.01; and ^#^*, p < 0.001*

To investigate the percentage of DNA damage within melanoma cells by the SN and RONS generated by CAP we performed a novel DNA damage study by evaluating γ-H2AX which binds specifically to DNA damage site using flow cytometry and immunocytochemistry (ICC). The percentage of γ-H2AX (DNA damage) produced by the co-treatment of the CAP and SN was increased more than 1.5 fold as compared to the control group (*p* < 0.01). However, the CAP-treated group showed nearly 1.5 fold γ-H2AX signals, comparable to the co-treated group (Fig. 3a). The number of γ-H2AX foci per cell was observed using ICC with DAPI staining showed that the co-treated group had significantly (*p* < 0.01) three times more H2AX foci as compared to the control (Fig. 3b, c).

A potent melanoma cell surface marker PD-1 and an enzyme DNA methyltransferase (DNMT) were also accessed to confirm the reduction of melanoma cells at the molecular level. The level of PD-1 is directly related to the number of melanoma cells and co-treatment group shows a decrease in PD-1 level as compared to the control (Fig. 3d). However, treatment with CAP alone did not show any effect on DNMT level and was comparable to the control (*p* > 0.05) (Fig. 3e), while synergistically, the effect of the CAP and SN significantly decreased the level of DNMT by two-fold (*p* < 0.01).

### Apoptosis induction molecular pathway

**Fig. 4:**
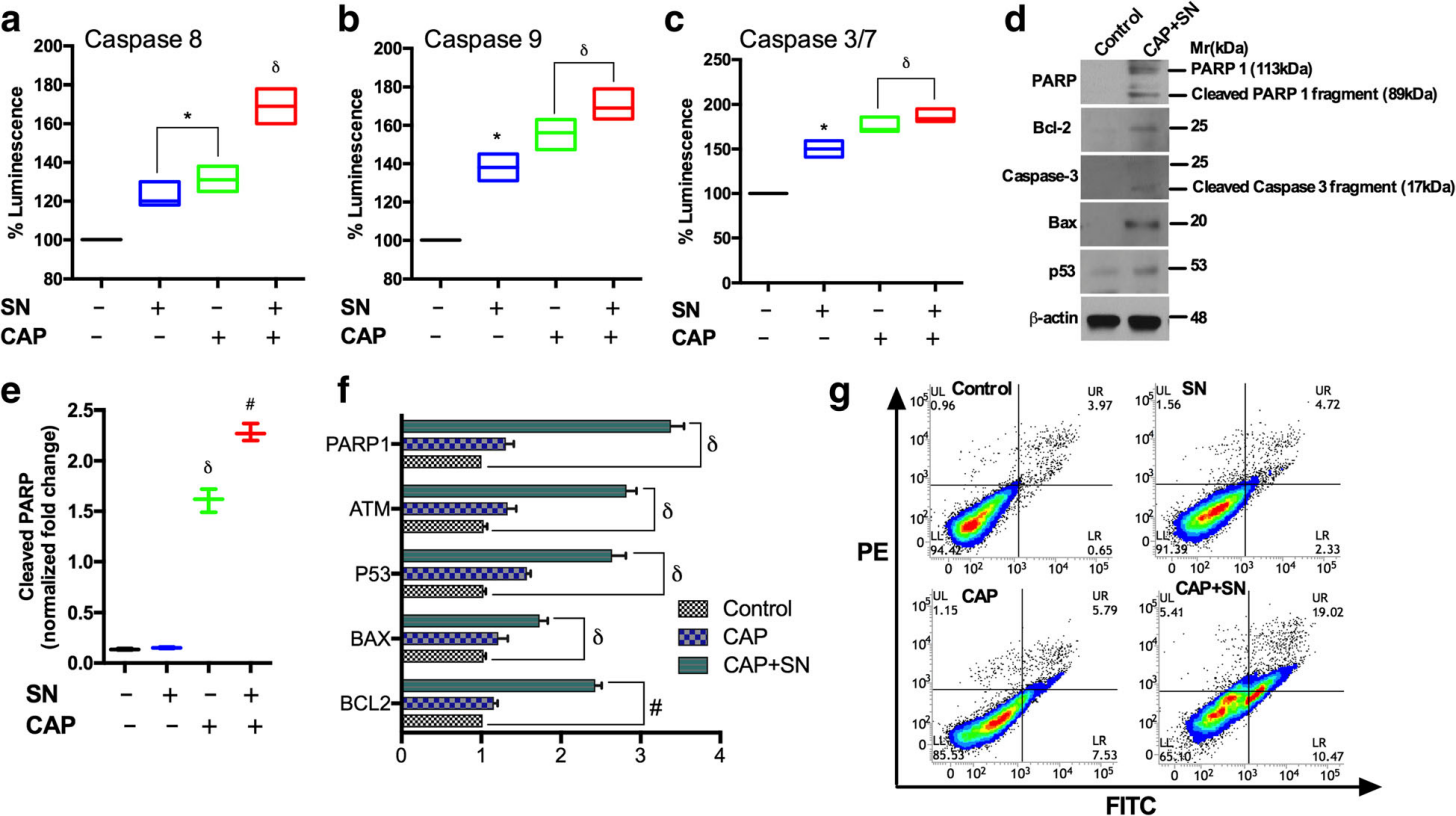
Co-inhibition by SN (100 nM) and CAP (180 s) in G-361 cellular death induction by different mechanisms. (**a**) Caspase 8, (**b**) caspase 9 and (**c**) caspase 3/7 levels by using spectrophotometrically (**d**) Immunoblot showing the detection of PARP/cleaved PARP, Bcl-2, caspase cleavage, Bax and the p53 activation status (**e**) Cleaved PARP estimation in different groups using spectrophotometrically. (**f**) The m-RNA expression levels of PARP-1, ATM, p53, BAX, and Bcl-2 were estimated using RT-PCR (**g**) Apoptotic cell death (annexin V/PI staining) was measured using flow cytometry. Error bars indicate the ±SD (*n* = 3). Student’s t-tests were performed, and the levels of significance are indicated as follows: ^*^, *p* < 0.05; ^δ^, *p* < 0.01; and ^#^*, p < 0.001*

The molecular damage was also estimated with the results showed a significant increase in cleaved PARP in the CAP-treated group (1.5 fold). In contrast, co-treated group showed a two-fold increase in the PARP activity as compared to the control and SN groups. However, SN alone did not show significantly cleaved PARP levels (Fig. 4e). The data also showed a significant increase in caspase levels in the co-treated group. Moreover, when using CAP and SN, caspase 3 and caspase 7 activity levels were increased by two-fold, while caspase 8 and 9 activity levels were increased by 1.5-fold (Fig. 4a, b, c). We assessed the expression levels of pro-apoptotic proteins after a CAP and SN treatment using western blotting and real-time PCR and confirmed the increased expression levels of the apoptotic genes of p53, ATM, BAX, Bcl-2 including PARP and caspase 3 in the co-treatment group (Fig. 4d, f)

Next, we also confirmed apoptosis using an Annexin V/PI apoptosis kit using flow cytometry. In this study, SN alone treatment showed only 4.72% apoptotic cells, comparable to that of the control sample (3.97%). However, CAP alone increased apoptosis marginally up to 5.79% while the number of apoptotic cells increased significantly by five times in the co-treated group as compared to the control group (Fig. 4g).

### Inhibition of the HGF/c-MET pathway signaling axis activates p53-mediated apoptosis in melanoma cells

**Fig. 5:**
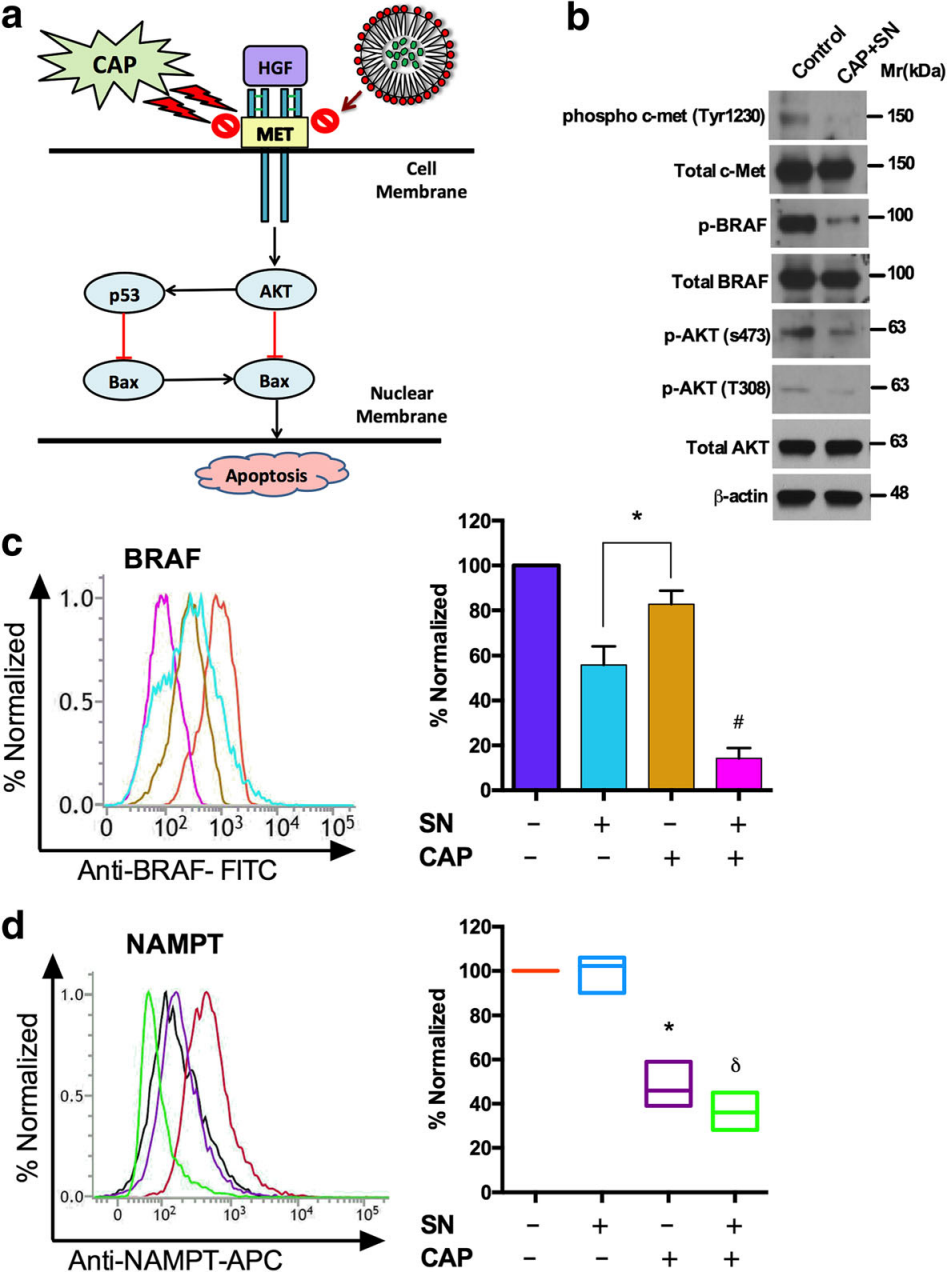
Dual effect of SN (100 nM) and CAP (180 s) assisting with the inhibition of the melanoma-specific HGF/c-MET pathway and its markers in G-361 cells. (**a**) Diagrammatic representation of the co-effect of SN and CAP to induce apoptosis of the HGF/c-MET pathway. (**b**) Western blot showing the phosphorylation and total status of c-MET and AKT at Tyr1230, S473 and T308 and phosphorylation and the total BRAF level (**c**) The melanoma-specific protein BRAF level and (**d**) Cellular NAMPT was calculated using a flow cytometer. Student’s t-tests were performed, and the levels of significance are indicated as follows: ^*^, *p* < 0.05; ^δ^, *p* < 0.01; and ^#^*, p < 0.001*

We proposed a combinational approach using CAP and SN to target the HGF/c-Met pathway in G-361 cells at effectively doses and checked the phosphorylation status of c-Met and its downstream axis (Fig. 5a). G-361 cells treated with CAP and SN showed a significant reduction in the expression levels of p-c-Met (Y1234/1235), BRAF and p-AKT (s473/T308) (Fig. 5b). Western blot results clearly supported our proposed pathway by inhibiting p-AKT, p-BRAF and p-cmet which leads to p53 mediated apoptosis. The BRAF is one of the important proto-oncogenes which gets mutated in case of melanoma and its lower expression attributed to the lowering of melanoma. The CAP alone has higher BRAF activity as compared to the SN group, but the co-treated group showed significantly reduced BRAF levels which signify a reduction in melanoma (Fig. 5c). NAMPT is a crucial enzyme is the key regulator of NAD synthesis which is implicated in the regulation of cellular metabolism and survival. The CAP alone and the co-treatment group showed a reduced level of NAMPT significantly upto 50% as as compared to the control group (Fig. 5d) attributable to the reduction in melanoma.

### Epithelial–mesenchymal transition (EMT) and cancer-stem-cell (CSC) maintenance downregulation within melanoma cells

**Fig. 6:**
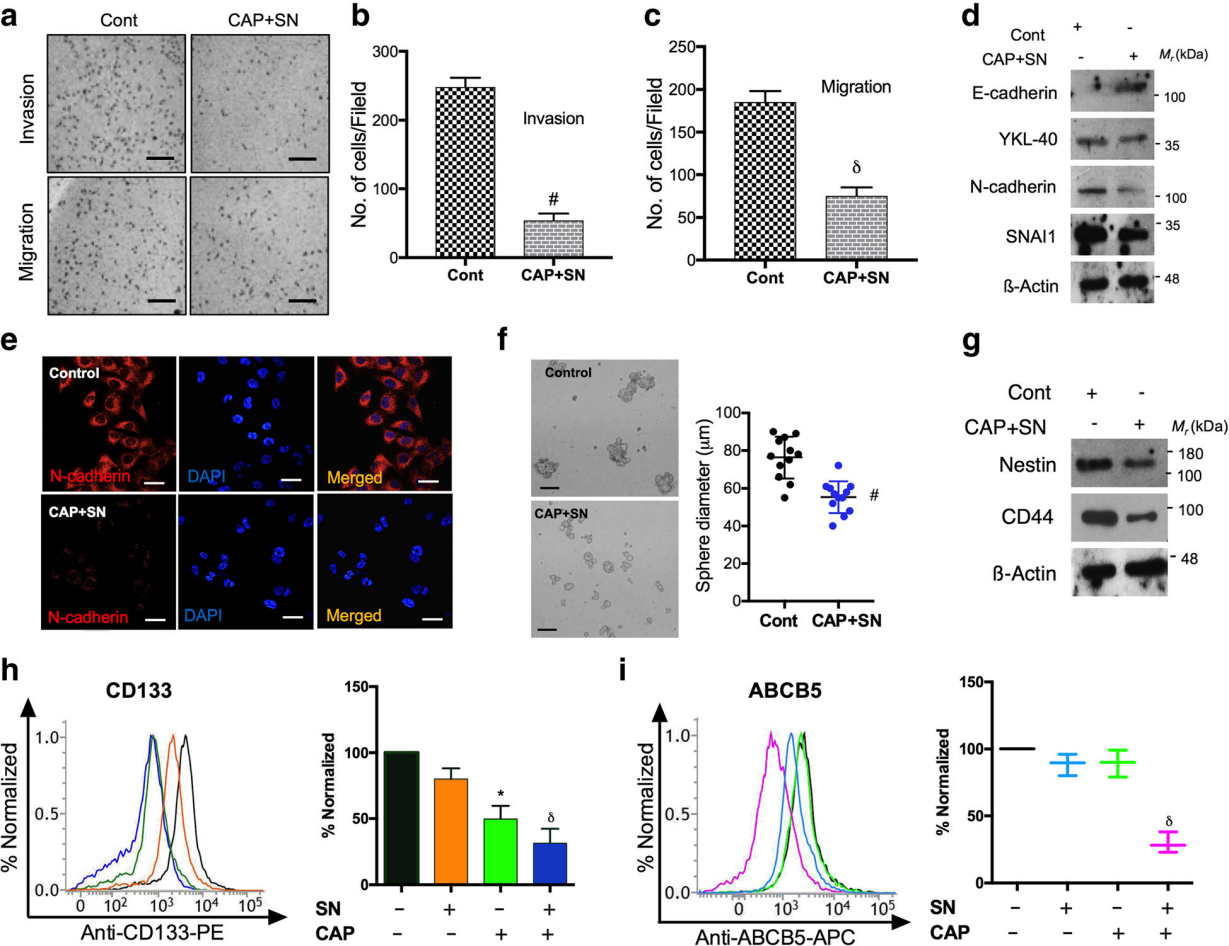
Bifold effect of SN (100 nM) and CAP (180 s) on the regulation of EMT and cancer stem cells in G-361 cells. (**a**) Migration and invasion assays were conducted after a co-treatment with 100 μM of SN and 180 s of CAP in G-361 cells. The scale bar corresponds to 30 mm. Graphical representation of G-361 cells that (**b**) invaded and (**c**) migrated under similar treatment conditions. (**d**) Western blot analysis of the EMT markers of E-cadherin, YKL-40, N-cadherin, and SNA/1 in G-361 cells (**e**) Immunocytochemistry of the mesenchymal marker N-cadherin in co-treated with G-361 cells and counterstained with DAPI. The scale bar corresponds to 10 mm. (**f**) Quantification of the sphere-forming ability of G-361 cells. The scale bar corresponds to 20 mm. (**g**) Western blot analysis of nestin and CD44 for the reduction in cancer stem cell markers (**h**) Flow cytometric analysis and graphical representation of CD133 expression levels after a co-treatment with SN and CAP using flow cytometry (**i**) Melanoma precise ABCB5 expression recorded in a flow cytometry analysis and its graphical representation using conditions identical to those above. Student’s t-tests were performed, and the levels of significance are indicated as follows: ^*^, *p* < 0.05; ^δ^, *p* < 0.01; and ^#^*, p < 0.001*

EMT is a characteristic feature of metastatic cancer and responsible for cellular invasiveness. CAP and SN combination treatment effectively downregulated the number of migratory events (~ 50%) and the invasive ability of G-361 cells (~ 46%) (Fig. 6a-e). A loss of mesenchymal markers such as YKL-40, SNAl1 and N-cadherin and gain in the epithelial marker E-cadherin were observed (Fig. 6d). Immunofluorescence data also confirmed our observations that N-cadherin expression levels (red color) were reduced after the CAP and SN combination treatment in G-361 cells (Fig. 6e).

A recently proposed model postulates that EMT can generate cancer stem-like cells (CSCs) and express many CSCs markers which play main roles in tumor relapse and metastasis [15–17]. A marked reduction in the sphere size in G-361 sphere cells treated with the CAP and SN combination were observed (Fig. 6f). CD44 and nestin expression levels (CSCs markers), were also downregulated after the co-treatment as compared with control (Fig. 6g). Subsequently, we evaluated decrease in expression of stem cell surface markers (CD133 and ABCB5) in combination group as compared to the outcomes for the groups treated with CAP and SN alone (Fig. 6h, i).

### Inhibition of tumorigenesis in mice model

**Fig. 7:**
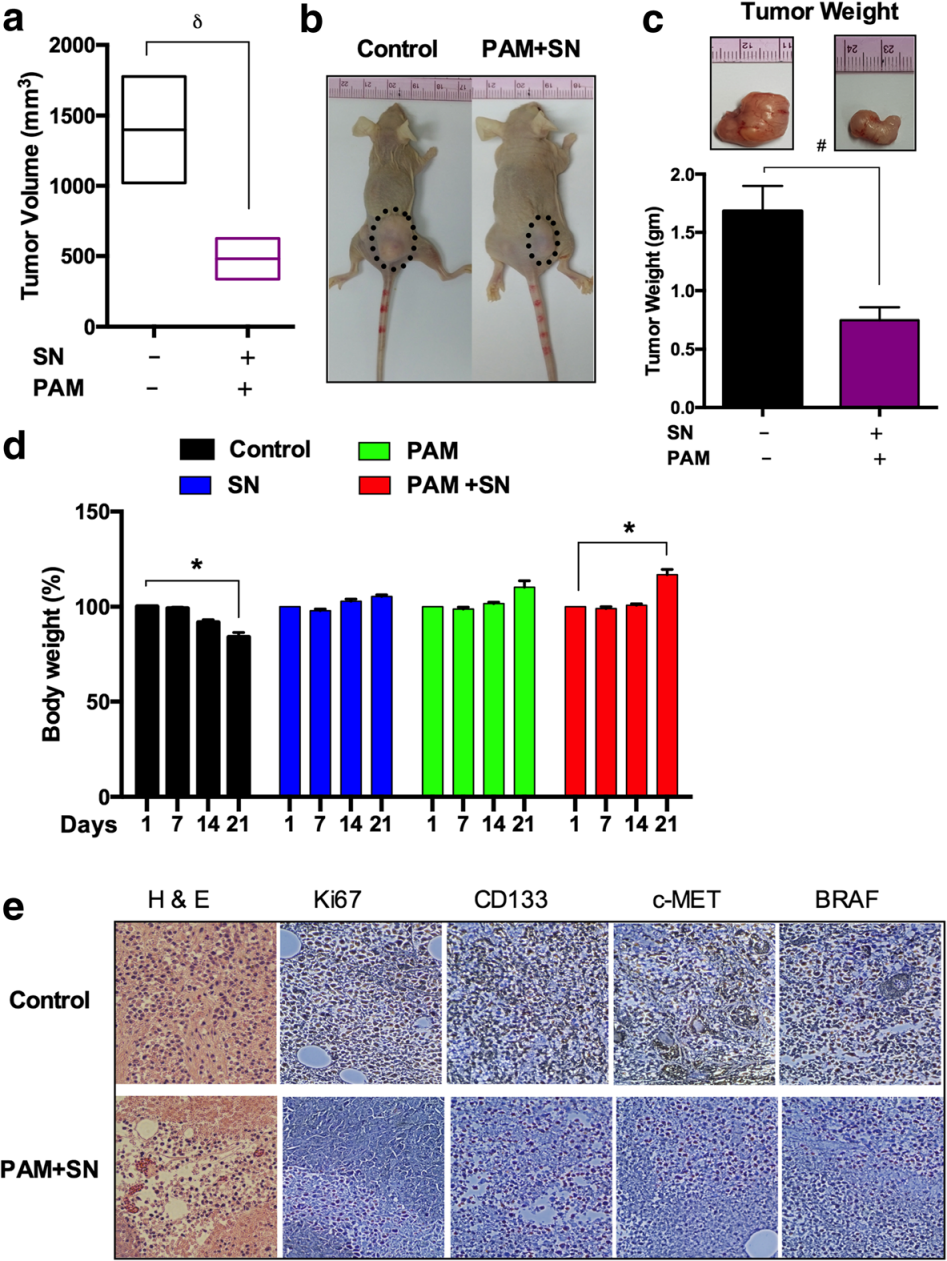
Anti-tumor effect of SN and PAM treatment in mice model. (**a**) Changes of the tumor volume in xenograft mice models. (**b**) Macroscopic observation of a control group and CAP-activated media and SN treated nude mice group bearing subcutaneous tumors on the right hind flank. (**c**) The bar graph shows the mean tumor weight of the excised control and treated melanomas. Photographs of solid melanomas removed from control and treatment groups at the time of the termination of this study are shown above the respective bar graphs. (**d**) Graphical representation of body weights (%) of the control and treated nude mice groups from 1 day to 21 days. (**e**) Representative hematoxylin and eosin (H & E) staining of tissue sections of both groups (magnification, 40X). Immunohistochemical analysis of Ki67, CD133, C-MET and BRAF expression levels in treated and untreated tumors tissues. The scale bar corresponds to 100 mm. Student’s t-tests were performed, and the levels of significance are indicated as follows: ^*^, *p* < 0.05; ^δ^, *p* < 0.01; and ^#^*, p < 0.001*

To explore more about inhibitory effect on melanoma we checked the effect of SN and PAM on animal model. After injecting G-361 cells intradermally the animal received SN and PAM co-treatment which decreased the tumor volume as compared to the untreated control group (Fig. 7a, b). The SN and CAP only group didn’t showed much reduction in tumor (data not shown) but SN and PAM co-treatment resulted in a 50% reduction of the tumor weight as compared to the control (Fig. 7c). Animal body weights in the control group decreased at 14 and 21 days, whereas in the combined treatment group, it was elevated at later intervals (Fig. 7d). Additionally, histological differences in the tumor sections between the SN and PAM co-treated and untreated groups were checked. Nuclei were found to be highly condensed (apoptotic cell death) in the co-treated groups, as compared to untreated controls observed using hematoxylin stain. In addition, immunohistochemical staining revealed that BRAF, c-MET, CD133 and Ki-67 levels were abolished compared to those in the control group (magnification at 40X) (Fig. 7e).

## Discussion

Melanoma is one of the most malignant cancers in human with a strong tendency for metastases if untreated in its earlier stages. Therefore, increasing interest has been focused on the search of a technique which is easily accessible, safe and effective against melanoma. The applications of CAP in the cancer treatment are almost new and its anti-cancer mechanism is very limited. Also, nanotechnology plays a crucial role in anti-cancer mechanism so we take both CAP and nanosilymarin for the reduction efficacy of human melanoma in in vitro and in vivo. In this study, a low concentration of SN (100 μM) enhanced the effects of non-thermal μ-DBD CAP (180 s) on melanoma cells and induced cellular toxicity, RONS, and apoptosis at non-toxic levels. This was attributed due to the presence of a liquid cell culture medium layer that facilitates the transition of reactive oxygen and nitrogen species from the gas phase to the dissolved liquid phase [18] during CAP treatment. The group treated with the CAP and SN revealed reduced metabolic viability in a time-dependent manner according to metabolic viability assays and also showing some degree of toxicity at later time intervals. This principal reason for this is the generation of free radical generated within the liquid surface of the culture medium after CAP treatment in combination of SN. Another plausible reason is that the short-lived free radicals at later time-intervals may convert into inactive radical which could react with the component of cell media/SN and formed non radical species which may be responsible for cell death. CAP generates many high reactive free radicals and charged particles that can self-react or interact with the medium, leading to additional RONS production [4]. These short-lived ROS interact with the components of the culture medium, leading to the production of long-lived reactive hydroperoxides [19]. The high level of RONS is attributed to the generation of very stable nitrite (NO_2_^−^) and nitrate (NO_3_^−^) ions which accumulate in melanoma cells, leading to the formation of intermediates such as *NO_2_, N_2_O_3_ or *NO, which cause nitration and nitrosation of important biological macromolecules such as DNA, RNA, proteins and lipids and alter their functions [20]. Previous studies have shown that intracellular RONS can induce apoptosis through increased caspase activity [21] that serve as the primary mediators of apoptosis, possibly allowing PARP upregulation to decrease melanoma progression. This is a plausible reason for the apoptosis in G-361 cells due to the synergistic effect of CAP. In addition, phosphorylation of H2AX leads to the apoptosis after the CAP and SN treatment, indicating that the induction of DNA damage is mediated through the induction of apoptotic pathway [22]. Moreover, the inability of the cells to repair the induced DNA damage may ultimately lead to the induction of apoptosis, as observed by annexin V-PI and p53 activation. PD-1 blockade is now becoming standard for many cancer conditions, as it helps to keep T cells from attacking other cells in the body [23]. Its expression is directly related to melanoma induction, with the results showing that the CAP and SN co-stimulatory effect reduced PD-1 expression levels in G-361 cells, leading to melanoma induction and providing evidence of a useful treatment strategy for melanoma patients having high PD-1 expression levels [24]. Another study found that DNA methylation induces tumor-suppressor genes through promoter hyper-methylation and aberrance in melanoma [25] which was estimated by DNMT. The significant reduction in DNMT enzyme indicates hypomethylation of G-361 cells in the co-treatment group signifies a low frequency of melanoma. Given that the CAP and SN co-treatment induced cellular damage in G-361 melanoma cells, we subsequently sought to determine the factors that could be responsible for CAP and SN induced cellular damage. To achieve the same previously, it was suggested that PARP inhibition damages the repair of endogenous single-strand breaks (SSBs), which could be converted into double-strand breaks (DSB) during the DNA replication process [26]. It was reported that ROS generated due to oxidative stress leads to DNA lesions [27]. ROS also leads to The increase in cleaved or activated PARP in the co-treatment group signifies target melanoma cells with defects in the double-strand DNA repair mechanism with subsequent cellular Caspases, particularly caspase 3 and 7, cleave the 116-kDa form of PARP-1 to generate an 85- and a 24-kDa fragment, on which we performed luminescence studies using ELISA for estimations of different caspases (caspase 8,9,3/7) [28]. These outcomes also signify that the induction of caspases occurs due to the proteolytic activity of caspase 9 through the phosphorylation of the protein at Ser-136 [29]. These results support our observations that cellular damage by caspases and PARP leads to the induction of apoptosis, as proved by an annexin-V/PI experiment.

Several reports have indicated that the overexpression and hyper-activation of c-Met (receptor tyrosine kinase) when it binds to its ligand hepatocyte growth factor (HGF), play an important role in melanoma development [30]. The efficacy of c-Met, mTOR, and AKT inhibitor combinations on resistant melanoma cells was tested in vitro, and a combination of all three inhibitors was found to be very effective [31]. Mutational activation of BRAF is the most prevalent genetic alteration in human melanoma [32] and the CAP and SN co-treatment demonstrated the suppression of oncogenic BRAF during melanoma maintenance. Recently, a melanoma-specific NAMPT marker has been utilized to diagnose tumoral cytokine, which is released and overexpressed in melanoma cells, and to promote proliferation and inhibit p53-dependent apoptosis in human melanoma cells [33]. Also, NAMPT is the key enzyme for NAD which is the source of energy in many pathways. Decrease in NAMPT enzyme directly leads to blockage of the energy pathways in G-361 cells and indicates melanoma reduction. c-Met signaling induces a reprograming network and benefits cancer-stem-cell-like phenotypes. c-Met hyperactivation increases tumorigenicity and tumor cell resistance to agents which damage DNA, also demonstrating properties associated with tumor-initiating stem cells. Melanoma metastasis is followed by the EMT phenomenon, as observed through a loss of cell adhesion and an increase in the mesenchymal phenotype [34]. Interestingly, dual targeting by the CAP and SN treatment effectively suppressed the mesenchymal transition and maintenance of tumor-initiating or stem-like cells which are generated through EMT. Stem cells renewal and differentiation plays a crucial role for normal development. However, OH^•^, NO_2_^−^ and NO_3_^−^ radicals formed because of oxidative stress within cell culture media leads DNA and protein damage in stem cells which leads to its reduction [35]. Our results showed reduction in CD133 and ABCB5 markers in CAP and SN co-treated group which signifies the damage in melanoma stem cells. Using a melanoma xenograft model, we demonstrated that melanoma cells are more sensitive to the SN and PAM treatment, as evidenced by decreased tumor size and growth. Interestingly, PAM contains extra free radicals due to the combination of free radical bombardment from CAP with cell culture media upper surface. This leads to the generation of free radical cascades and formation of OH^−^, NO_2_^−^, H_2_O_2_ etc. which combined with the SN and may leads to non-radical development which leads to inhibit tumor size growth and weight.

## Conclusion

In summary, we suggest that blocking HGF signaling with SN and CAP can overcome the EMT phenomenon in melanoma cells. Ongoing efforts include testing the SN with PAM in mice xenografts showed promising results. Together these outcomes provide a rationale to use the SN to improve the efficacy of CAP with a targeted drug-delivery system as part of melanoma treatment approaches and for this system to serve as a therapeutic target for melanoma patients in the future.

## Additional file

Additional file 1: **Figure S1.** Total cellular damage effects of the SN and CAP treatment of SK-MEL-5 cells. (TIF 133 kb)

## Abbreviations

CAP: Cold atmospheric plasma
DAF-FM-DA: 4-amino-5-methylamino-2′,7′-difluorofluorescein diacetate
DNMT: DNA Methyl transferase
EMT: Epithelial-mesenchymal transition
H_2_DCFDA: 2,7-dichloroflurodiacetate
HGF/c-MET: Hepatocyte growth factor/cellular- Mesenchymal to Epithelial Transition
MTT: 3-(4,5- dimethyl thiazol − 2-Yl)-2,5-diphenyltetrazolium bromide
PAM: Plasma activated Media
PARP: Poly (ADP-Ribose) Polymerase
PD-1: rogramme cell death protein-1
PI: Propidium iodide
q-PCR: Quantitative real-time polymerase chain reaction
SN: Silymarin nanoemulsion
μ-DBD: micro-dielectric barrier discharge
